# Supplementary material for: Fluorescence In Situ Hybridization (FISH) Tests for Identifying Protozoan and Bacterial Pathogens in Infectious Diseases
Source: Diagnostics (Basel). 2022 May 21;12(5):1286. doi: 10.3390/diagnostics12051286 (PMC9141552; doi:10.3390/diagnostics12051286)
Supplement: Supplementary file 1 [file diagnostics-12-01286-s001.zip › Supplementary Figure S2. Procedure for TB FISH.pdf]

Liquid culture

Add Sample Processing Buffer (1:1),
5-10min room temp, add MycoSPR Buffer (8:1), mix

Solid culture

Add a loopful of bacteria to water
and glass beads, mix by vortexing

Make each smear with 10 µl of processed culture

Air dry

Fix with phenol/ethanol 5 min

Air-dry

Pre-treatment

15 min 37°C

Rinse 1x in Pre-treatment Rinse buffer

Air-dry

Add 10 µl Hybridization Buffer with probes, cover with cover-slip

15 min 37°C

Wash with 1x wash buffer, 3x

Air-dry in dark

Add mounting medium and cover with cover-slip

View at x1000 magnification in microscope with LED/filter attachment
