# Supplementary figures and images for: Fluorescence In Situ Hybridization (FISH) Tests for Identifying Protozoan and Bacterial Pathogens in Infectious Diseases
Source: Diagnostics (Basel). 2022 May 21;12(5):1286. doi: 10.3390/diagnostics12051286 (PMC9141552; doi:10.3390/diagnostics12051286)

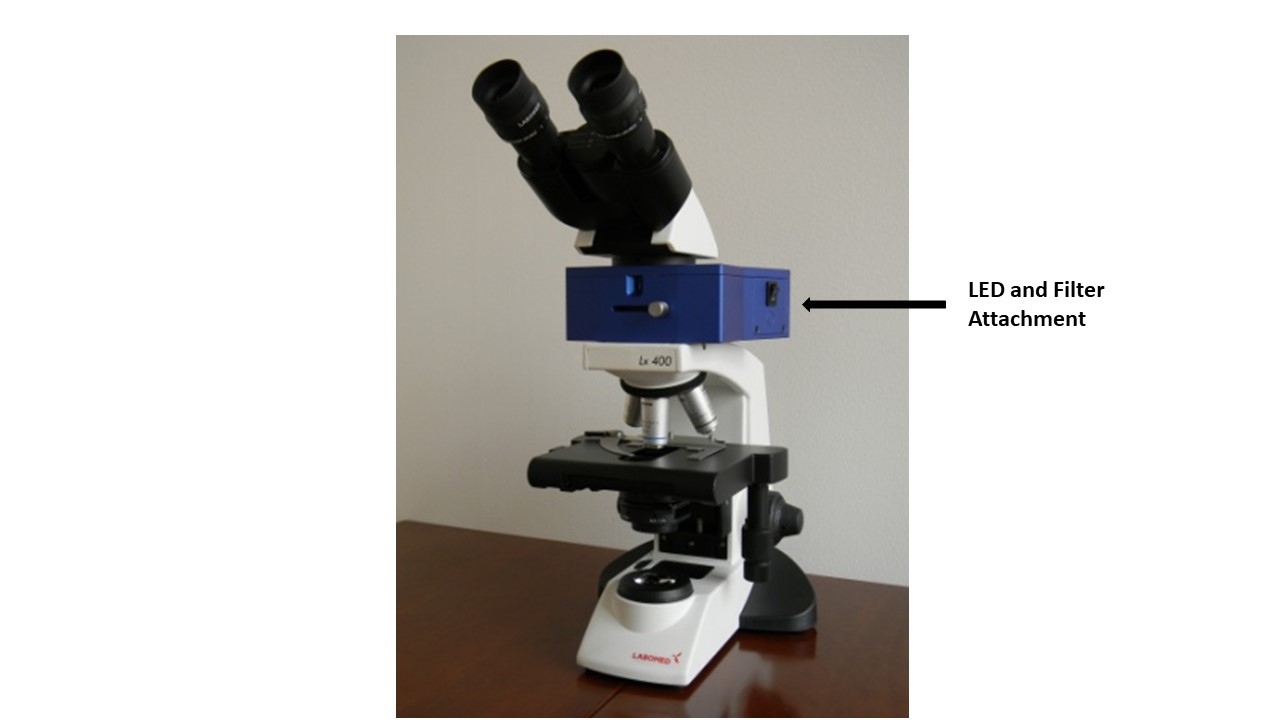

Supplement: Supplementary file 1 [file diagnostics-12-01286-s001.zip › Supplementary Figure S1 Microscope with LED & filter attachment.jpg]
